# Supplementary material for: Spatial autocorrelation and epidemiological survey of visceral leishmaniasis in an endemic area of Azerbaijan region, the northwest of Iran
Source: PLoS One. 2020 Aug 26;15(8):e0236414. doi: 10.1371/journal.pone.0236414 (PMC7449399; doi:10.1371/journal.pone.0236414)
Supplement: Attachment — Submitted filename: Response to Reviewers.docx [file pone.0236414.s003.docx]

PONE-D-19-35837
Spatial Autocorrelation and Epidemiological Survey of Visceral Leishmaniosis in an endemic area of Azerbaijan Region, Northwest of Iran
PLOS ONE

Additional Editor Comments (if provided):

-Improve titles in Results section to be more descriptive. ( Ok , Edited )

- Include legends to figures. (Ok , Edited )

- Check figures axes titles. For example, Y axis in figure 4 is labeled ‘Vl’ instead of ‘VL’. Ok , Edited )

-. Please ensure you have thoroughly discussed any potential limitations of this study within the Discussion section. Ok, the limitations of the study were discussed at the end.

Journal Requirements:

When submitting your revision, we need you to address these additional requirements:

1. Please ensure that your manuscript meets PLOS ONE's style requirements, including those for file naming. The PLOS ONE style templates can be found at http://www.plosone.org/attachments/PLOSOne_formatting_sample_main_body.pdf and <http://www.plosone.org/attachments/PLOSOne_formatting_sample_title_authors_affiliations.pdf>

(Ok, was done )

2. In the ethics statement in the manuscript and in the online submission form, please provide additional information about the patient records used in your retrospective study. Specifically, please ensure that you have discussed whether all data were fully anonymized before you accessed them and/or whether the IRB or ethics committee waived the requirement for informed consent. If patients provided informed written consent to have data from their medical records used in research, please include this information. (Ok, was done )

3. Please ensure you have thoroughly discussed any potential limitations of this study within the Discussion section. (Ok, was done )

4. During your revisions, please note that a simple title correction is required to ensure correct spelling. The title should read: "Spatial Autocorrelation and Epidemiological Survey of Visceral Leishmaniasis in an endemic area of Azerbaijan Region, Northwest of Iran". Please ensure this is updated in the manuscript file and the online submission information. (Ok, was done )

5. Thank you for stating the following in the Acknowledgments Section of your manuscript:

"This work was supported by the Ardabil University of Medical Sciences (Project numbers 92218)."

We note that you have provided funding information that is not currently declared in your Funding Statement. However, funding information should not appear in the Acknowledgments section or other areas of your manuscript. We will only publish funding information present in the Funding Statement section of the online submission form.

Please remove any funding-related text from the manuscript and let us know how you would like to update your Funding Statement. Currently, your Funding Statement reads as follows:

"Ardabil University of Medical Sciences " (Ok, was done )

6. We note that you have indicated that data from this study are available upon request. PLOS only allows data to be available upon request if there are legal or ethical restrictions on sharing data publicly. For information on unacceptable data access restrictions, please see http://journals.plos.org/plosone/s/data-availability#loc-unacceptable-data-access-restrictions.

No , data not in available.

In your revised cover letter, please address the following prompts:

a) If there are ethical or legal restrictions on sharing a de-identified data set, please explain them in detail (e.g., data contain potentially identifying or sensitive patient information) and who has imposed them (e.g., an ethics committee). Please also provide contact information for a data access committee, ethics committee, or other institutional body to which data requests may be sent.

b) If there are no restrictions, please upload the minimal anonymized data set necessary to replicate your study findings as either Supporting Information files or to a stable, public repository and provide us with the relevant URLs, DOIs, or accession numbers. Please see http://www.bmj.com/content/340/bmj.c181.long for guidelines on how to de-identify and prepare clinical data for publication. For a list of acceptable repositories, please see http://journals.plos.org/plosone/s/data-availability#loc-recommended-repositories.

We will update your Data Availability statement on your behalf to reflect the information you provide.

7. We note that Figures #1, 6 and 7 in your submission contain map images which may be copyrighted. All PLOS content is published under the Creative Commons Attribution License (CC BY 4.0), which means that the manuscript, images, and Supporting Information files will be freely available online, and any third party is permitted to access, download, copy, distribute, and use these materials in any way, even commercially, with proper attribution. For these reasons, we cannot publish previously copyrighted maps or satellite images created using proprietary data, such as Google software (Google Maps, Street View, and Earth). For more information, see our copyright guidelines: http://journals.plos.org/plosone/s/licenses-and-copyright.

We require you to either (1) present written permission from the copyright holder to publish these figures specifically under the CC BY 4.0 license, or (2) remove the figures from your submission:

1.    You may seek permission from the original copyright holder of Figures #1, 6 and 7 to publish the content specifically under the CC BY 4.0 license.

*** I propped figures 1, 6 and 7 with the Arcmap GIS 10.4.1 software and uploaded the original file of GIS.*****

We recommend that you contact the original copyright holder with the Content Permission Form (http://journals.plos.org/plosone/s/file?id=7c09/content-permission-form.pdf) and the following text:

“I request permission for the open-access journal PLOS ONE to publish XXX under the Creative Commons Attribution License (CCAL) CC BY 4.0 (http://creativecommons.org/licenses/by/4.0/). Please be aware that this license allows unrestricted use and distribution, even commercially, by third parties. Please reply and provide explicit written permission to publish XXX under a CC BY license and complete the attached form.”

Please upload the completed Content Permission Form or other proof of granted permissions as an "Other" file with your submission.

In the figure caption of the copyrighted figure, please include the following text: “Reprinted from [ref] under a CC BY license, with permission from [name of publisher], original copyright [original copyright year].”

2.    If you are unable to obtain permission from the original copyright holder to publish these figures under the CC BY 4.0 license or if the copyright holder’s requirements are incompatible with the CC BY 4.0 license, please either i) remove the figure or ii) supply a replacement figure that complies with the CC BY 4.0 license. Please check copyright information on all replacement figures and update the figure caption with source information. If applicable, please specify in the figure caption text when a figure is similar but not identical to the original image and is therefore for illustrative purposes only.

The following resources for replacing copyrighted map figures may be helpful:

USGS National Map Viewer (public domain): http://viewer.nationalmap.gov/viewer/

The Gateway to Astronaut Photography of Earth (public domain): http://eol.jsc.nasa.gov/sseop/clickmap/

Maps at the CIA (public domain): https://www.cia.gov/library/publications/the-world-factbook/index.html and https://www.cia.gov/library/publications/cia-maps-publications/index.html

NASA Earth Observatory (public domain): http://earthobservatory.nasa.gov/

Landsat: http://landsat.visibleearth.nasa.gov/

USGS EROS (Earth Resources Observatory and Science (EROS) Center) (public domain): http://eros.usgs.gov/#

Natural Earth (public domain): http://www.naturalearthdata.com/

8. Please include a copy of Table 2 which you refer to in your text on page 6 and Table 1 and 3 which you refer to in your text on page 7.

Ok , was done

Reviewer #1: Dear Authors
Please consider the following comments:

1- Please double-check the spelling of the words in all manuscript. For example in title please change leishmaniosis to leishmaniasis. ( Ok , it is done)

2- The article needs to be edited in English by a native.( It was native).

3- Please define in methodology how did you calculated the incidence of VL?

R: (The effect of variables, such as age, sex and location, on the prevalence (No. of VL cases/population size * 100000) of the disease was evaluated using SPSS version 23(Chicago, IL, USA)

4- Please explain more about the method of analysis for Moran's Index and General G analysis. Did you used the points of disease or considered the counties as the analysis units.

R: (Moran's I Index and General G value were measured, and both the score and p-values (p < 0.05) were calculated and used to evaluate the significance of the index (27). Moran’s I is a commonly used indicator of spatial autocorrelation. In this study, global Moran’s I was used as the first measure of spatial autocorrelation. Its values range from−1 to 1. The value “1” means perfect positive spatial autocorrelation (high values or low values cluster together), while “−1” suggests perfect negative spatial auto-correlation (a checkerboard pattern), and “0” implies perfect spatial randomness (28, 29) . The High/Low Clustering (General G) tool measures how concentrated the high or low values are for a given study area. This tool calculates the High/Low General G value (observed & expected) & the associated Z score & p-value for a given input feature class(30).)

5- Please cite a relevant reference for MaxEnt model.( Ok , Reference 31 , Young N, Carter L, Evangelista P. A MaxEnt model v3. 3.3 e tutorial (ArcGIS v10). Fort Collins, Colorado. 2011.)

6- The reference 24 cited in line 104 seems to be irrelevant to the analysis.( Reference edited )
7- Fig 5 for the result of spatial auto-correlation should be changed with a map resulted from this analysis. (Figure 5 is the software output and is intended to illustrate the relationship).

8- In all maps please use "Urmia Lake" , ( Ok , Was done).

9- Table 1 was not included in the reviewed file. ( This is for Editor)

10- Please discuss more about the role of weather and environmental variables on the habitat suitability for life cycle of VL.

Reviewer #2: This study analyzes the spatial patterns of visceral leishmaniasis in three provinces (Ardabil, East Azerbaijan and West Azerbaijan) in northwestern Iran from 2009 to 2017, as well as its relationship with environmental and geographical factors. The manuscript has a relevant theme, but needs an important review/organization in its content and writing. ( Ok , The manuscript was edited native )

-I suggest a comprehensive review of the English written in the manuscript (typographical / grammatical errors and scientific writing); Ok , The manuscript was edited native

-Please describe clearly and comprehensively the main problem/object addressed in the manuscript; The introduction was edited

-The introduction does not provide an entirely relevant description of the proposal and significance of the study. The problem is not significantly and concisely stated in the text; ( Ok , Accordingly, this study aimed to investigate the geographical distribution of VL in three provinces of Ardabil, East Azerbaijan and West Azerbaijan (northwestern Iran) and determine the high-risk areas of the disease in the provinces and its relation with environmental and geographical factors. Another objective of the study was to determine the environmental suitability for VL in northwestern Iran for prevention and control of diseases.)

-The methods needs to include some additional methodological details in the text (e.g., how morbidity data was extracted or made available; case definition; more information from the surveillance system responsible for the data, etc.), as well as a comprehensive and detailed description of all analyzes presented in the results; Ok, was edited ((In this retrospective cross-sectional study, the Data on VL cases were collected from Ardabil, East Azerbaijan and West Azerbaijan Provinces health centers during the last 9yr from January 2009 to the end of December 2017. The number of patients with Direct Agglutination Test (DAT) positive serologic tests was more than 1:3200 cases with medical records, diagnosed by physicians and treated in different parts of the province. By referring to health centers in different counties and reviewing patients’ hospital and health records, demographic characteristics, including age, sex, place of residence, month and year of the disease as well as the results of the DAT test were collected. Then, the data collected were compared with the general statistics in the offices of the vice-chancellor for the health of the provinces of East Azerbaijan, West Azerbaijan, and Ardabil, and the results of the comparison were recorded. The collected data were stored in an ArcMap environment based on the patients’ addresses.))

-Overall, the results are not clearly presented and the interpretations and conclusions are not supported and justified by the results, but rather general statements. The limitations of this study are not presented in the text (e.g., coverage and quality of secondary data); Once again the whole article was edited according to your comments and it was native.

-The titles of figures and tables are not provided in the manuscript, and the tables were not included in the main file.
