# Supplementary material for: Spatial autocorrelation and epidemiological survey of visceral leishmaniasis in an endemic area of Azerbaijan region, the northwest of Iran
Source: PLoS One. 2020 Aug 26;15(8):e0236414. doi: 10.1371/journal.pone.0236414 (PMC7449399; doi:10.1371/journal.pone.0236414)
Supplement: Attachment — Submitted filename: Response to Reviewers.docx [file pone.0236414.s004.docx]

Additional Editor Comments (if provided):

1. Introduction, line 43. I suggest adjusting ´there is a high correlation between the life cycle of the VL disease and the environmental factors involved in it ...´ to  ´there is a high correlation between the occurrence of the VL disease and the environmental factors involved in it ...´. Ok, was done.
2. Methods, lines 111-114. Please, refer to table 1 here: ´A total of 19 climate variables were downloaded from the 111 Worldclime website (www.worldclime.com) with a resolution of 30 sec (≅1 sq.km) and were used 112 along with an elevation variable at the same resolution to evaluate and determine the appropriate 113 ecological niches (Table 1)´. Ok, was done.
3. Tables 2 and 3. Please, invert the tables. Titles are correct, but the corresponding tables have been inverted. Ok, was done.
4. Table ´The results of T-test and Kruskal –walls test for incidence of VL ...´. In the cell ´<1000 -1150>´, I suggest substituting this label with either ´Lower than 1000 and higher than 1150´, or ´<1000 and >1150´. Ok, was done.
5. Results, lines 175-176. Please, further clarify the sentence ´ the results of the investigation of the impact of all variables together on the model were precipitation seasonality (35.40%) and mean diurnal range (21.60%)´. As currently written it is confusing. Ok, was done and edited (Once, the impact of each variable on the model was evaluated separately (Fig 8). In addition, the impact of all variables together on the model were precipitated again. The model showed that two factors; seasonality (35.40%) and mean diurnal range (21.60%) had the greatest impact on the occurrence of the disease)
6. Results, line 176. Please, correct ´(Table 1and 3)´ to (Table 1)´. Ok, was done.

Reviewer #1: Dear Authors
Thank you for addressing most of the comments. Still I think two comments are not addressed:
1- Did you used the points of VL disease for Moran's analysis or considered the counties as the analysis units? We used the points of VL disease for Moran's analysis.

2- Fig 5 for the result of spatial auto-correlation should be replaced with a map resulted from this analysis. You can ask Arcmap to plot the map i this analysis. To determine the autocorrelation, the Moran and G index have been used, and the Arcmap software output is in the form of a graph, not a map.

Best regards

Dr.Eslam Moradi-Asl
